# Age-Related Changes in Risky Decision Making and Associated Neural Circuitry in a Rat Model

**DOI:** 10.1523/ENEURO.0385-22.2022

**Published:** 2022-01-11

**Authors:** Caitlin A. Orsini, Wonn S. Pyon, Richard J. Dragone, Mojdeh Faraji, Alexa-Rae Wheeler, Marjory Pompilus, Marcelo Febo, Jennifer L. Bizon, Barry Setlow

**Affiliations:** 1 Department of Psychiatry, University of Florida, Gainesville, Florida 32610; 2 Department of Neuroscience, University of Florida, Gainesville, Florida 32610; 3 McKnight Brain Institute, University of Florida, Gainesville, Florida 32610

**Keywords:** aging, decision making, neuroimaging, punishment, rat, risk taking

## Abstract

Altered decision making at advanced ages can have a significant impact on an individual’s quality of life and the ability to maintain personal independence. Relative to young adults, older adults make less impulsive and less risky choices; although these changes in decision making could be considered beneficial, they can also lead to choices with potentially negative consequences (e.g., avoidance of medical procedures). Rodent models of decision making have been invaluable for dissecting cognitive and neurobiological mechanisms that contribute to age-related changes in decision making, but they have predominantly used costs related to timing or probability of reward delivery and have not considered other equally important costs, such as the risk of adverse consequences. The current study therefore used a rat model of decision making involving risk of explicit punishment to examine age-related changes in this form of choice behavior in male rats, and to identify potential cognitive and neurobiological mechanisms that contribute to these changes. Relative to young rats, aged rats displayed greater risk aversion, which was not attributable to reduced motivation for food, changes in shock sensitivity, or impaired cognitive flexibility. Functional MRI analyses revealed that, overall, functional connectivity was greater in aged rats compared with young rats, particularly among brain regions implicated in risky decision making such as basolateral amygdala, orbitofrontal cortex, and ventral tegmental area. Collectively, these findings are consistent with greater risk aversion found in older humans, and reveal age-related changes in brain connectivity.

## Significance Statement

Changes in cost–benefit decision making at advanced ages can be modeled in rats. Although such models have largely corroborated age changes in some forms of decision making, they have not evaluated decisions involving risks of adverse consequences (i.e., punishment). The current study evaluated young and aged rats in a decision making task involving risk of punishment. As in humans, aged rats were more risk averse than young rats. This greater risk aversion was accompanied by greater functional connectivity among brain regions implicated in risky decision making. The findings suggest that greater risk aversion in aging is due to inherent (biological) factors and establish a model in which future work can evaluate neural mechanisms underlying age-related changes in risk taking.

## Introduction

The ability to make effective decisions is critical for managing finances, health care, and other activities necessary to maintain personal independence. There is growing appreciation, however, that decision making can change at advanced ages, and that such changes may negatively impact life quality, health, and well being in older adults (Denburg et al., 2007; Boyle et al., 2012). In comparison with fully mature young adults, healthy older adults tend to make less impulsive and less risky choices (although the way in which risks are framed can influence both the extent and direction of age differences; Green et al., 1996, 1999; Deakin et al., 2004; Jimura et al., 2011; Löckenhoff et al., 2011; Mata et al., 2011; Samanez-Larkin et al., 2011; Eppinger et al., 2012; Liebherr et al., 2017; Wilson et al., 2021). Such age-related reductions in impulsivity and risk taking are often considered beneficial; however, they also have the potential to be maladaptive (e.g., when they contribute to the avoidance of necessary medical procedures or promote conservative financial strategies that forgo expenditures necessary to maintain quality of life). Understanding the behavioral and neural underpinnings of age-related changes in decision making could yield insights into how such maladaptive decision strategies arise and how they might be addressed.

A challenge in studying decision making across the life span is that it may be affected by environmental factors that covary with age, such as income or experiences with significant cultural and political events (Green et al., 1996). These challenges can be largely circumvented through studies in rodents, however, in which environmental variables can be tightly controlled. For example, aged rats exhibit less impulsivity (greater preference for large, delayed rewards over small, immediate rewards) compared with fully mature young adults (Simon et al., 2010; Roesch et al., 2012; Hernandez et al., 2017, 2019), which is consistent with findings in human subjects (Green et al., 1996, 1999; Samanez-Larkin et al., 2011; Eppinger et al., 2012; but see Seaman et al., 2022). In contrast, aged rats show few differences compared with young adults on tasks involving risks of reward omission (choices between small, guaranteed and large, probabilistic rewards; Gilbert et al., 2011; Samson et al., 2015). The relative absence of age differences in these tests of risk taking runs counter to findings in humans (Cauffman et al., 2010; Mata et al., 2011; Josef et al., 2016; Mata et al., 2016); however, the possibility of reward omission models only one aspect of risky decision making (choices involving probabilistic reward) and not others that are equally important (choices involving probabilistic punishment). The latter can be assessed using a “risky decision making task” (RDT), in which rats make discrete choices between the following two options: one that yields a small, “safe” food reward and another that yields a large food reward accompanied by varying probabilities of mild footshock punishment (Simon et al., 2009; Orsini et al., 2019b). Behavior in this task corroborates several aspects of human risk-taking behavior, including sex differences, relationships with drug self-administration, and neurobiological correlates (Mitchell et al., 2014; Orsini et al., 2015a, 2016, 2018). As such, the primary goal of the current study was to determine how advanced age affects decision making under risk of punishment in the RDT.

All forms of decision making require integration of multiple cognitive operations that may decline with age and contribute to alterations in decision making (James et al., 2015). In aged humans, greater discounting of delayed rewards (greater impulsive choice) is associated with worse episodic memory retrieval (Lempert et al., 2020). Similarly, worse executive functioning among older adults is associated with less risk taking on some behavioral tasks (Wilson et al., 2021) but greater risk taking in others (Brand and Schiebener, 2013; Sinclair et al., 2021). Among aged rats, greater preference for large, delayed rewards over small, immediate rewards (less impulsive choice) is associated with better working memory but worse cognitive flexibility, despite aged rats as a group being impaired on both measures (Hernandez et al., 2017). Among young adult rats, greater risk taking in the RDT predicts greater cognitive flexibility in a set-shifting task (Shimp et al., 2015). As such, a secondary goal of the current study was to determine whether age changes in RDT performance are associated with cognitive/motivational alterations that could contribute to risk-taking behavior.

Adaptive decision making involves coordinated activity among multiple brain regions (Floresco et al., 2008; Orsini et al., 2015b, 2019a; Piantadosi et al., 2021). Neural activity in many of these structures changes across the life span (Tomasi and Volkow, 2012; Sala-Llonch et al., 2015; Lighthall, 2020), but the relationships between such changes and age-related alterations in decision making are unclear. Consequently, a third goal of this study was to evaluate age differences in functional connectivity using functional magnetic resonance imaging (fMRI), with a particular focus on brain regions implicated previously in decision making under risk of punishment [several prefrontal cortical subregions, nucleus accumbens shell (NAcSh), amygdala, and ventral tegmental area (VTA); Simon et al., 2011; Mitchell et al., 2014; Orsini et al., 2015a, 2017, 2018; Pyon et al., 2021].

## Materials and Methods

### Subjects

Young adult (6 months of age, *n* = 20) and aged (24 months of age, *n* = 18) male Fischer 344 × Brown Norway F1 hybrid (FBN) rats (National Institute on Aging colony maintained by Charles River) were individually housed and kept on a 12 h light/dark cycle (lights on at 7:00 A.M.) with access to water and food *ad libitum*, except as noted in experimental procedures. Rats were housed in the vivarium in the McKnight Brain Institute building at the University of Florida and were tested in three cohorts, each with at least *n* = 6 young rats and *n* = 3 aged rats. Before the start of behavioral testing, rats were habituated to handling by the experimenters over several days. During behavioral testing, rats were food restricted to 85% of their free-feeding weight to encourage participation in the tasks. Behavioral testing was conducted on weekdays between 10:00 A.M. and 3:00 P.M. All animal procedures were conducted in accordance with the University of Florida Institutional Animal Care and Use Committee and followed the guidelines of the National Institutes of Health.

### Behavioral testing apparatus

Behavioral testing was conducted in 12 operant test chambers (Coulbourn Instruments), housed in sound-attenuating cabinets. Each chamber contained a food pellet delivery trough equipped with a photobeam to detect entries and a 1.12 W lamp to illuminate the trough, into which 45 mg soy-free food pellets (TestDiet; 5TUM) could be dispensed. The trough was centrally located in the front of the chamber and ∼2 cm above the floor. Each chamber contained two retractable levers 11 cm above the floor on both the left and right sides of the food trough. An additional 1.12 W house light was mounted to the top of the rear wall of the cabinets. The floor of each chamber was composed of a row of stainless steel bars coupled to a shock generator (Coulbourn Instruments), which was used to administer scrambled footshocks. An activity monitor was mounted on the ceiling of the chamber to detect locomotor activity through the use of an array of infrared detectors. The test chambers were controlled via Graphic State 4.0 software (Coulbourn Instruments), which allowed programmable steps for each task protocol and collection of data from the operant chambers. In each cohort, the order in which young and aged rats were tested in each chamber was counterbalanced, and chambers were cleaned with dilute chlorhexidine between successive rats.

### Behavioral procedures

#### Risky decision making task

Shaping procedures were designed to train rats to reliably press the two response levers to earn food pellet rewards (Orsini et al., 2018, 2021). Shaping began with magazine training, during which single food pellets were delivered into the food trough with an intertrial interval of 100 ± 40 s, for a total of 38 deliveries. Criterion performance for magazine training was 100 nosepokes into the food trough in a 64 min session. Rats were then trained to press one of the two levers (left or right, counterbalanced across ages), which delivered single food pellets on a fixed ratio 1 schedule. The passing criterion for lever press training was 50 presses in a 30 min session. After reaching this criterion on one lever, rats were then shaped to press the other lever under the same criterion. Rats were subsequently trained in discrete trials to nosepoke into the food trough on trough light illumination. On each trial, a nosepoke in the food trough triggered extension of either the left or right lever (randomly chosen within each pair of trials), a press on which resulted in a single food pellet. Following the lever press, the lever retracted and the trough light was extinguished. Criterion performance was defined as a total of 30 presses on each lever within a 60 min session.

**Figure 1.**
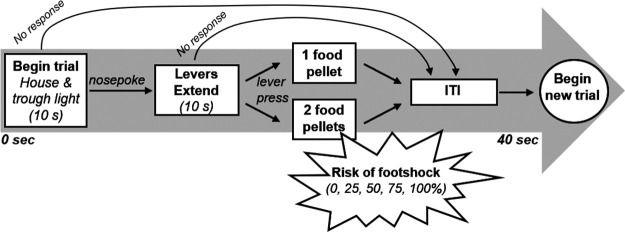
Schematic of the risky decision making task. Each 40 s trial is initiated with the illumination of the house and food trough lights. A rat must nosepoke into the food trough to trigger the extension of one lever (forced-choice trials) or both levers (free-choice trials). A press on the small, safe lever yields 1 food pellet, delivered immediately. A press on the large, risky lever yields 2 food pellets, also delivered immediately, but accompanied by a varying probability of footshock delivery (0, 25, 50, 75, and 100%). After an intertrial interval ranging from 10 to 35 s (depending on how quickly the rat progresses through the trial components), a new trial begins.

The design of the RDT is illustrated in Figure 1. This task was designed to assess the preference of rats for a small, safe reward (one food pellet) versus a large, “risky” reward (two food pellets) that is accompanied by varying probabilities of mild electrical footshock (Simon et al., 2009; Orsini et al., 2019b). Rats began testing in the RDT immediately following the completion of shaping. Each session of the RDT was 60 min in duration and consisted of five 18-trial blocks. Each 40 s trial began with illumination of both the house and food trough lights. A nosepoke into the food trough extinguished the trough light and resulted in extension of either a single lever (forced-choice trial) or both levers (free-choice trial). If a rat did not nosepoke within 10 s, both lights were extinguished and the trial was counted as an omission. A press on the small, safe reward lever always resulted in delivery of a single food pellet, whereas a press on the large, risky reward lever always resulted in delivery of two food pellets, but was also accompanied by a mild footshock (1 s, 200–300 μA; shock intensities differed across the three cohorts, but remained constant within each cohort), the probability of which was specific to each block. The probability of footshock accompanying a large lever press was set at 0% for the first block of trials and increased by 25% across each subsequent block (0, 25, 50, 75, and 100%, respectively). The large food reward was always delivered on choice of the large, risky lever, regardless of whether a shock was delivered. The left/right position of the small versus large reward levers was counterbalanced across age groups; however, for each rat, these positions remained consistent throughout testing. Each block of trials began with 8 forced-choice trials in which the punishment probabilities were established (four presentations of each lever, randomly presented), which were followed by 10 free-choice trials. On the forced-choice trials (which were designed to remind rats of the shock probabilities in effect for that block), the probability of footshock delivery on a large lever press was dependent across the four forced trials for this lever. For example, during the 25% block, one and only one of the four forced-choice trials on which the large reward was delivered resulted in shock delivery; in contrast, during the 75% block, three and only three of the four forced-choice trials on which the large reward was delivered resulted in shock delivery. Unlike forced-choice trials, the probability of shock on each free-choice trial was independent of outcomes of other free-choice trials (e.g., such that each press of the large reward lever in the 25% block had a one in four chance of triggering a footshock, regardless of shock deliveries on previous trials in that block). Each cohort of rats was trained on the task until stable performance was attained (see Data Analysis section below).

#### Shock reactivity threshold testing

After completion of testing in the RDT, a shock reactivity threshold was determined for each rat to evaluate potential age differences in how rats perceive/respond to the shocks (Orsini et al., 2021). For this test (which took place in an operant chamber in a different room from that used for the other behavioral tasks), each rat was initially exposed to a shock of 400 μA (0.5 s) to reduce spontaneous locomotor activity in the test chamber. The shock was then set to 50 μA (0.5 s) and delivered every 30 s, with the shock amplitude increasing by 25 μA with each successive shock until a flinch response (usually sharp withdrawal of a single paw) was observed. After a flinch response was elicited, the shock was decreased in 25 μA increments until no response was observed, and then once again increased until a response was observed. This pattern continued until at least three flinches were observed and the last shock delivery did not result in a flinch. The average of the three shock intensities at which flinches were detected was considered to be the shock reactivity threshold and was used in data analysis.

#### Probabilistic reversal learning task

After shock reactivity threshold testing, rats were tested on a probabilistic reversal learning task to evaluate cognitive flexibility (Dalton et al., 2014; Hernandez et al., 2021). Each 50 min session (conducted in the same operant chambers used for the RDT) consisted of 200 choice trials with a 15-s intertrial interval. Each trial began with illumination of the house light, followed 3 s later by extension of both levers into the chamber. At the start of each session, one of the two levers was selected at random as “correct,” and the other as “incorrect.” A press on the correct lever resulted in delivery of a single food pellet with a probability of 0.80. A press on the incorrect lever resulted in delivery of a single food pellet with a probability of 0.20. If the rat failed to press either lever within 10 s of lever extension, the trial was considered an omission, and both levers were retracted and the house light extinguished. If the correct lever was chosen for eight consecutive trials, the correct and incorrect levers were switched, such that the lever that was previously rewarded at 0.80 now was rewarded at 0.20 and vice versa. This pattern was repeated for the duration of the 200 trials. Rats were trained on the probabilistic reversal learning task for eight consecutive sessions.

#### Progressive ratio task

The progressive ratio task was conducted in the same operant chambers used for the RDT and reversal learning tasks. This task was designed to evaluate the motivation of rats to work to obtain food rewards and has been used previously in aged rats (Hernandez et al., 2017). A single lever was extended into the chamber (the same lever designated as the “small, safe” lever in the RDT) and remained extended for the duration of the session. At the start of each session, the first lever press yielded a single food pellet, and, as the session progressed, the number of presses required to earn pellets increased on each trial using the following sequence: 1, 4, 10, 20, 35, 56, 84, 120, 165, and on ( $N\text{ = }\frac{\text{(}r\text{(}r\text{ + 1)(}r\text{ + 2))}}{\text{6}}$, where *N* is the number of presses required to earn a reward *r* and where *r* is the ordinal number of the reward; for example, for the second reward, *r *=* *2; for the third reward, *r *=* *3). Sessions were terminated when 10 min had elapsed since the last reward was delivered. Rats were tested on the progressive ratio task for seven consecutive sessions.

### Behavioral data analysis

Raw data files were compiled using Graphic State 4.0 software and organized in Microsoft Excel using custom macros. The organized data were analyzed using SPSS 27.0. Choice performance in each block of the RDT was measured as the number of trials on which the large reward was chosen. For the purposes of statistical analyses, these data were arcsine transformed to account for imposing an artificial ceiling (10/block) on the number of possible large reward lever presses (Winstanley et al., 2004). To determine stable behavioral performance on the RDT, a two-factor repeated-measures ANOVA was conducted on free-choice trials across three consecutive sessions, with session and trial block as within-subject factors and age as a between-subjects factor. Stable performance was defined as the absence of a main effect of session and no interaction between session and trial block (Simon and Setlow, 2012). Effects of age on free-choice trials (averaged across the three consecutive sessions of stable performance) were determined using a two-factor repeated-measures ANOVA, with trial block as the within-subject factor and age as the between-subjects factor. The Greenhouse–Geisser correction was applied in cases in which homogeneity of variance was violated. In addition to analyzing choice performance across blocks, trial-by-trial analyses were conducted to determine whether the outcome of previous trials affected subsequent choice differently between young and aged rats. To do this, win–stay and lose–shift behavior were compared between young and aged rats. Win–stay behavior, which provided a measure of sensitivity to rewarding outcomes, was calculated by dividing the number of trials on which a rat chose the large, risky lever after receiving the large reward without shock delivery by the total number of trials on which a rat received the large reward without shock delivery. In contrast, lose–shift behavior, which provided a measure of sensitivity to negative feedback (i.e., punishment), was calculated by dividing the number of trials on which the rat chose the small, safe lever after receiving both the large reward and shock delivery by the total number of trials on which both the large reward and shock were delivered. These behavioral measures were analyzed with a two-factor repeated-measures ANOVA, with trial type (win–stay vs lose–shift) as the within-subjects factor and group as the between-subjects factor.

Additional performance measures in the RDT, including response latency (time between lever extension and lever press) on forced-choice and free-choice trials, trial omissions, and locomotor activity during both intertrial intervals and shock delivery, were also analyzed. Latencies for each forced-choice trial block were averaged across the 3 d of stable performance and then analyzed with a three-factor repeated-measures ANOVA, with lever identity and trial block as within-subjects factors and age as a between-subjects factor. Similarly, latencies for each free-choice trial block were averaged across the 3 d of stable performance; however, because some rats chose one lever exclusively in three or more blocks (e.g., rats only pressed the small, safe lever in the 75% and 100% trial blocks), there were insufficient data to include trial block as a within-subjects factor in a repeated-measures ANOVA. Consequently, latencies for each lever were averaged across the five trial blocks. These mean response latencies were then analyzed with a two-factor repeated-measures ANOVA, with lever identity as the within-subjects factor and age as the between-subjects factor. Trial omissions and locomotor activity (either during intertrial intervals or shock delivery) were analyzed using an independent-samples *t*-test in which age was included as the between-subjects factor.

Shock threshold values, as determined in the shock reactivity threshold assay, were compared between young and aged rats using an independent-samples *t*-test.

In the probabilistic reversal learning task, the number of successful completed reversals per session (multiplied by 200 and then divided by the number of trials completed to account for trial omissions) was compared between young and aged rats across the 8 d of testing using a two-factor repeated-measures ANOVA, with session as a within-subjects factor and age as a between-subjects factor. Trial-by-trial analyses were also performed to determine whether young and aged rats differed in how they used misleading (e.g., no reward after a correct choice) feedback to guide subsequent choice. For this task, win–stay behavior (correct choice after a rewarded correct choice) was calculated by dividing the number of trials on which a rat chose the correct lever after a rewarded correct choice by the total number of rewarded correct choices. Conversely, lose–shift behavior (incorrect choice after a nonrewarded correct choice) was calculated by dividing the number of trials on which a rat chose the incorrect lever after a nonrewarded correct choice by the total number of nonrewarded correct choices. Win–stay and lose–shift behavior were averaged across the 8 d of this task and subjected to a two-factor repeated-measures ANOVA, with trial type (win–stay vs lose–shift) as the within-subjects factor and age as the between-subjects factor.

To further explore performance in the probabilistic reversal learning task, choice behavior (i.e., selection of correct vs incorrect lever) and individual learning rates were modeled and estimated used the following Rescorla–Wagner equation (Rescorla and Wagner, 1974):

(1) $$V_{t+1}^{k}=V_{t}^{k}+\alpha \left(r_{t}-V_{t}^{k}\right),$$

where V is the associative value of choice k to reward, r is the reward, and α is the learning rate, which has a value between 0 and 1, with 0 indicating no learning and 1 indicating instant learning. Reward value, $r_{t}$, is equal to 1 in trial t when a reward is received, and equal to 0 if no reward is earned. Associative value, V, is set to zero at the beginning of each session when no information from the environment has yet been acquired, and is updated using Equation 1 as trials proceed. Choice behavior was modeled using a softmax function (Eq. 2) that converts the associative values to action probabilities, as follows:

(2) $$P_{t}^{k}=\frac{\text{exp}(\beta V_{t}^{k})}{\sum_{i=1}^{K}\text{exp}(\beta V_{t}^{i})},$$

where $p_{t}^{k}$ is the probability of choice k in trial t. Parameter β is the inverse temperature, which represents the stochasticity of choice behavior and ranges from 0 to positive infinity, with 0 indicating random behavior and positive infinity indicating a fully deterministic choice of the highest-value option. The initial value of the probabilities is $P_{0}^{L}=P_{0}^{R}=0.5$ for both the left and right lever since both have similar initial associative values $V_{0}^{L}=V_{0}^{R}=0$. Learning rates and the inverse temperature parameter are estimated for each animal and each session by maximizing the logarithm of the likelihood function (Eq. 3), as follows:

(3) $$\left\{\hat{\alpha ,}\hat{\beta }\right\}=\underset{\alpha \in \left[0,1\right],\beta \in [0,\infty )}{\text{arg max}}\ln\;{\hat{L}}_{n}\left(\alpha ,\beta ;C\right),$$

where L is the likelihood function, *C* is the vector of choice behavior, and n is the number of trials. L, is computed as a product of Bernoulli trials (Eq. 4), as follows:

(4) $$L_{n}\left(\alpha ,\beta ;\text{C}\right)=\overset{n}{\underset{t=1}{\prod }}\left(c_{t}^{L}P_{t}^{L}+c_{t}^{R}P_{t}^{R}\right),$$

where the Bernoulli outcome is the choice behavior, and probabilities are calculated using the softmax function (Eq. 2). Outcome, $c_{t}^{k}$, is equal to 1 if chosen in trial t, and equal to 0 if not chosen.

Finally, in the progressive ratio task, the number of lever presses, the number of rewards earned, and the ratio at which the rats ceased pressing (i.e., their breakpoint) were each averaged across the seven sessions. An independent-samples *t*-test was used to compare these values between young and aged rats.

For all analyses, if there were significant main effects or interactions in a multifactor parent ANOVA, additional *post hoc* ANOVAs were used to determine the source of the significance. Values of *p *≤* *0.05 were considered statistically significant. Effect sizes are reported with η^2^ for ANOVAs, and with Cohen’s *d* for *t*-tests.

Spearman’s correlations were used to examine relationships between risky choice and performance in the probabilistic reversal learning task, progressive ratio task, and shock reactivity threshold assay. Arcsine-transformed data were averaged across blocks 2 through 5 of the RDT (i.e., trial blocks in which risk of punishment was present) and the resulting value was used in these correlational analyses.

### Functional neuroimaging

#### Apparatus

Functional MRI data were collected using a 11.1 tesla Bruker MRI Scanner (Magnex Scientific) with an RRI BFG-240/120-S6 Integrated Gradient and Shim Coil System with a bore size of 120 mm, a maximum gradient strength of 1000 mT/m, and a rise time of 200 μs, which was operated using Bruker AV3 HD console software (Bruker). A quadrature transmit/receive radio frequency (RF) coil tuned to a 470 MHz 1H resonance was used for B1 field excitation and RF signal direction. Functional images were collected using one-shot spin-echo echoplanar imaging (EPI) sequence with the following parameters: echo time (TE) = 15 ms; repetition time (TR) = 2000 ms; field of view (FOV) = 25.6 × 25.6 mm^2^ in plane; 20 slices with 1.0 mm thickness; data matrix = 64 × 64. No stimuli were presented during functional scanning. Anatomical scans for image overlay and reference-to-atlas registration were collected using a fast spin echo sequence (TR/TE_eff_ = 2500/48 ms; rapid acquisition with relaxation element factor = 16; number of averages = 6; FOV = 25.6 × 25.6 mm^2^; 1.0 mm thick; data matrix = 256 × 256) in the same space as the EPI scan.

#### Magnetic resonance imaging

Two of the three cohorts of rats used for behavioral testing underwent neuroimaging procedures. Cohort 2 (*n* = 6, young; *n* = 3, aged) underwent imaging after testing in all of the behavioral tasks described above. Cohort 3 (*n* = 7, young; *n* = 5, aged) underwent neuroimaging immediately after testing in the RDT, as further behavioral testing was precluded by laboratory closure because of COVID-19.

Rats were imaged using isoflurane (1.5%) anesthesia. Spontaneous breathing rate was monitored during setup and MRI acquisition. Body temperature was maintained at 37°C using a warm water circulation tube system (SA Instruments). The head was stabilized using a bite bar and a series of foam pads to allow for optimal placement of the surface coil over the skull and to reduce any potential movement during scanning. Each rat underwent a 10 min high-resolution T2-weighted anatomic scan followed by a 10 min fMRI scan in the absence of any stimulation (“resting-state” conditions).

#### Image preprocessing

Processing of anatomic and functional scans was performed using custom-made UNIX bash scripts (available on request) calling functions and tools from the FMRIB Software Library (FSL version 6.0.3; Jenkinson et al., 2002), Analysis of Functional NeuroImages (AFNI; Cox, 1996), and Advanced Normalization Tools (ANTs; Klein et al., 2009). First, to segment relevant voxels containing brain structures from all other voxels, masks were manually generated using high-resolution anatomic scans uploaded to image segmenting software (ITK-SNAP; Yushkevich et al., 2006). The segmented anatomic scans were then aligned to Left-Posterior-Inferior orientation and registered to the Ferris MRI Rat Brain Atlas (Iriah et al., 2019) using FSL linear image registration tool (FLIRT; Jenkinson et al., 2002). Registration matrices for each rat were saved and used to transform fMRI datasets into atlas space for processing and analysis. Time series spikes in the fMRI scans were removed and displacements in individual frames and slice-timing delays were corrected using AFNI (3dDespike, 3dTshift, and 3dvolreg; Cox, 1996). fMR images were linearly registered to their corresponding segmented T2 anatomic scans using FSL FLIRT and warped to fit their respective anatomic scans. fMRI scans were merged into a single-image time series file from which white matter and ventricle signal were regressed out, followed by bandpass filtering (0.009–0.12 Hz) of the time series signal, spatial smoothing (1.2 mm FWHM), and voxel time series L2 normalization.

Brain regions of interest [ROIs; VTA, prelimbic cortex (PrL), NAcSh, lateral orbitofrontal cortex (lOFC), ventral orbitofrontal cortex (vOFC), basolateral amygdala (BLA)] were selected on the basis of data demonstrating their involvement in RDT performance (Simon et al., 2011; Mitchell et al., 2014; Orsini et al., 2015a, 2017, 2018). Bilateral masks of the ROIs were created using the Ferris Rat Brain Atlas. The masks were then used to extract average time series data from select ROIs from preprocessed fMRI scans. Time series data were then used to calculate bootstrapped Pearson *r* coefficients (*n* = 1000; *1dCorrelate* in AFNI) between pairs of ROIs.

#### Statistical analyses

Pearson *r* correlation coefficients were Fisher *z*-transformed (*FisherZ* in DescTools, version 0.99.38 of R) and entered into SPSS (version 28.0.0.0) for statistical analyses. A two-factor ANOVA with Bonferroni-adjusted *post hoc* tests was used to compare the functional connectivity of young and aged rats across ROI pairs. Arcsine-transformed data from the RDT were correlated with functional connectivity for each ROI pair (separately in young adult and aged rats, using Spearman’s ρ).

## Results

### Risky Decision making Task

**Figure 2.**
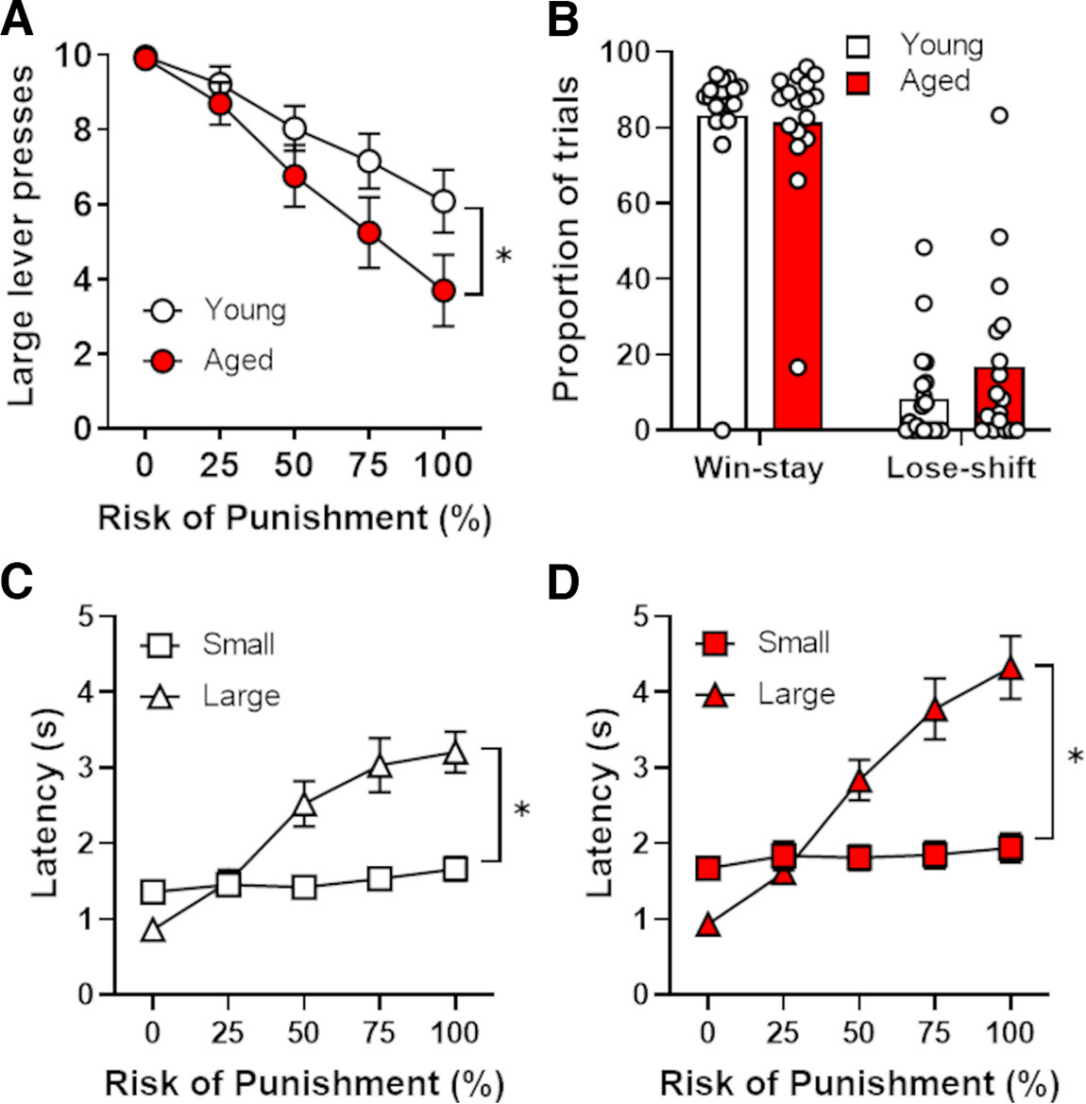
Performance on the Risky Decision-making Task in young and aged rats. ***A***, Aged rats made significantly fewer presses on the large, risky lever than young rats. ***B***, Aged and young rats displayed comparable win–stay and lose–shift behavior in the RDT. ***C***, Relative to latencies to press the small, safe lever, latencies to press the large, risky lever increased as the risk of punishment increased in young rats. ***D***, Relative to latencies to press the small, safe lever, latencies to press the large, risky lever increased as the risk of punishment increased in aged rats. Data are represented as the mean (±SEM) number of large lever presses (***A***), the proportion of trials (***B***), or the latencies in seconds (***C***, ***D***). *p ≤* *0.05.

Rats were trained in the RDT until choice performance was stable, which required 30–45 sessions of testing. A two-factor ANOVA (age × trial block) revealed the expected main effect of risk of punishment, such that rats reduced their choice of the large reward as the risk of punishment increased across blocks of trials (main effect of trial block: *F*_(4,144)_ = 34.47, *p *<* *0.01, η^2^ = 0.49; Fig. 2*A*). The main effect of age did not reach statistical significance (*F*_(1,36)_ = 3.23, *p *=* *0.08, η^2^ = 0.08), but there was a significant interaction between age and trial block, such that aged rats chose the large, risky lever less frequently than young rats as the risk of punishment increased across trial blocks (*F*_(4,144)_ = 3.23, *p *=* *0.05, η^2^ = 0.08). A two-factor repeated-measures ANOVA conducted on win–stay/lose–shift behavior revealed, however, that there was no main effect of age (*F*_(1,35)_ = 1.09, *p *=* *0.30, η^2^ = 0.03) or age × trial type (win–stay vs lose–shift) interaction (*F*_(1,35)_ = 0.95, *p *=* *0.34, η^2^ = 0.03; Fig. 2*B*). Hence, despite age differences in lever preference, aged and young rats appear to process outcome feedback comparably. Considered together, these data suggest that aged rats are more risk averse than young in the face of potential punishment.

In addition to lever preferences, the latencies of rats to press the levers on forced-choice and free-choice trials in each trial block were also assessed (excluding omitted trials) and were used as a measure of incentive motivation to obtain each reward (Giertler et al., 2003; Schoenbaum et al., 2003; Shimp et al., 2015). Four aged rats omitted all of the forced-choice trials in some blocks (five such blocks in total). So as to not have to exclude the data of these animals, a conservative extrapolation approach was taken by using their latencies from the prior trial block in the missing trial block. As expected, a three-factor, repeated-measures ANOVA (age × trial block × lever identity) revealed that latencies to press the large reward lever were longer than latencies to press the small reward lever (main effect of lever identity: *F*_(1,36)_ = 20.91, *p *<* *0.01, η^2^ = 0.37) and that latencies increased overall with the increase in risk of punishment (main effect of trial block: *F*_(4,144)_ = 92.06, *p* < 0.01, η^2^ = 0.72; Fig. 2*C*,*D*). There was also a significant lever identity × trial block interaction, such that latencies increased across trial blocks only on the large reward lever (*F*_(4,144)_ = 48.00, *p *<* *0.01, η^2^ = 0.57). Further, there was a main effect of age, such that aged rats had longer latencies to press levers overall compared with young rats (*F*_(1,36)_ = 6.09, *p *=* *0.02, η^2^ = 0.15). Although the pattern of latency differences across trial blocks appeared to differ between age groups (particularly for the large reward lever), neither the age × trial block (*F*_(4,144)_ = 2.35, *p *=* *0.09, η^2^ < 0.01) nor age × trial block × lever identity (*F*_(4,144)_ = 2.19, *p *=* *0.12, η^2^ = 0.06) interactions reached statistical significance. In contrast to response latencies during forced-choice trials, there was no main effect of age on latencies to press levers during free-choice trials (*F*_(1,25)_ = 1.38, *p *=* *0.25, η^2^ = 0.05). There was also no main effect of lever identity (*F*_(1,25)_ = 0.06, *p *=* *0.80, η^2^ < 0.01) nor an interaction between lever identity and age (*F*_(1,25)_ = 1.46, *p *=* *0.24, η^2^ = 0.06).

Additional measures of RDT performance were compared between young and aged rats (Table 1). Aged rats omitted significantly more trials than young rats (*t*_(36)_ = −2.36, *p *=* *0.03, *d* = −0.80), but there were no age differences in locomotor activity, either during the intertrial intervals (*t*_(36)_ = 1.51, *p *=* *0.14, *d* = 0.48) or during shock delivery (*t*_(35)_ = 0.85, *p *=* *0.40, *d* = 0.28; note that data are missing from one rat who never chose the risky lever and thus never received shocks).

**Table 1:** Mean (±SEM) baseline locomotor activity, locomotor activity during shock delivery, and omissions in the risky decision making task

	Locomotor activity (locomotor units/ITI)	Shock reactivity (locomotor units/shock)	Omissions
Young	9.68 (2.04)	2.02 (0.27)	1.50 (0.67)*
Aged	6.07 (1.25)	1.71 (0.23)	7.74 (2.56)*

ITI, Intertrial interval.

*A main effect of age (*p *≤* *0.05).

### Shock reactivity threshold testing

**Figure 3.**
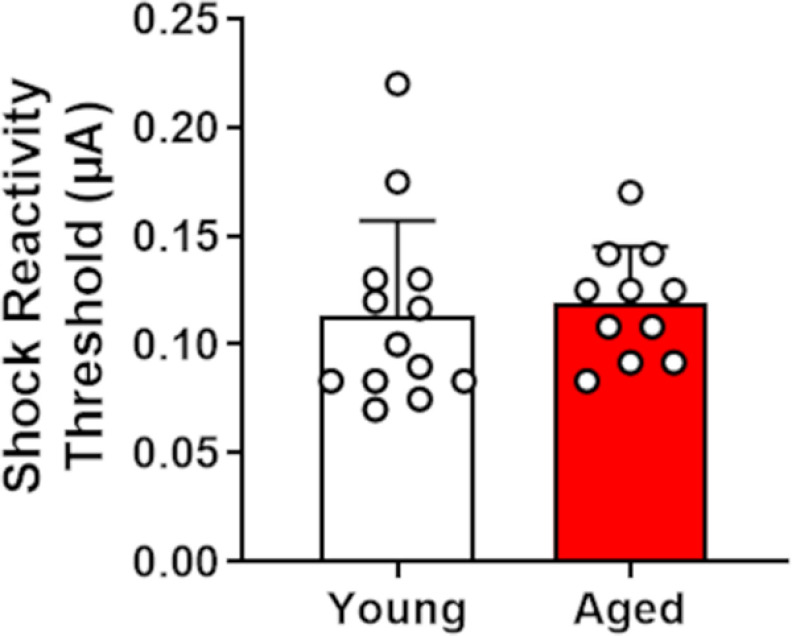
Performance on a shock reactivity threshold assay in young and aged rats. Shock thresholds were not significantly different between young and aged rats. Data are represented as the mean (±SEM) shock reactivity threshold (in μA).

To determine whether the age difference in RDT performance could be attributed to differential sensitivity to footshock, a subset of rats (*n* = 13 young rats; *n* = 11 aged rats; from Cohorts 1 and 2 only) was tested for their shock reactivity thresholds. An independent-samples *t*-test revealed no difference between age groups in their shock reactivity thresholds (*t*_(22)_ = −0.40, *p *=* *0.69, *d* = −0.17), suggesting that the reduced preference of aged rats for the large, risky reward was not because of greater sensitivity to the footshock punishment (Fig. 3).

### Probabilistic reversal learning task

**Figure 4.**
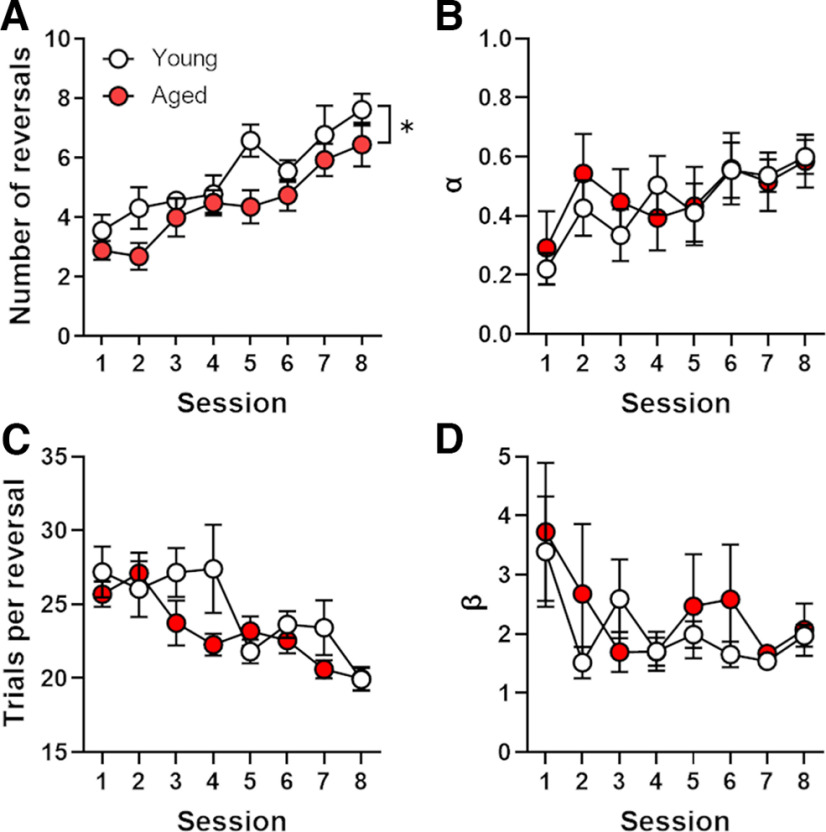
Performance on the probabilistic reversal learning task in young and aged rats. ***A***, Aged rats completed fewer reversals over the eight sessions of testing compared with young rats. ***B***, Data from the probabilistic reversal learning task were used to model the choice behavior of rats and to estimate learning rates. Learning rates significantly increased across the eight sessions of testing similarly in young and aged rats. ***C***, The mean number of trials per block decreased across the eight sessions of testing similarly in young and aged rats. ***D***, There was a significant decrease in stochasticity of choice behavior across the eight sessions of training that was evident in both young and aged rats. Data are represented as the mean (±SEM) number of reversals (***A***) or the learning rate parameters (***B–D***). *p ≤* *0.05.

Following shock reactivity threshold testing, rats (*n* = 13 young rats; *n* = 10 aged rats; from Cohorts 1 and 2 only) were tested in the probabilistic reversal learning task. A two-factor, repeated-measures ANOVA (age × session) compared the number of successful reversals per session (corrected for trial omissions) between age groups. This analysis revealed a main effect of session (*F*_(7,147)_ = 13.56, *p *<* *0.01, η^2^ = 0.27) such that rats increased the number of reversals attained per session (Fig. 4*A*). Although the interaction between age and session was not significant (*F*_(7,147)_ = 0.77, *p *=* *0.61, η^2^ = 0.02), aged rats made fewer reversals than young rats across sessions (main effect of age: *F*_(1,21)_ = 4.20, *p *=* *0.05, η^2^ = 0.05), indicating that reversal learning is modestly impaired in aging. Importantly, both young and aged rats completed almost all trials in the reversal learning task, with no age difference in the percentage of omitted trials [mean (SEM) percentage of omitted trials: young rats, 0.47 (0.22); aged rats, 1.54 (0.80); *t*_(21)_ = −1.29, *p *=* *0.22, *d* = −0.61]. Despite the fact that aged rats made fewer reversals than young rats, both groups used feedback from correct choices (rewards vs no rewards) comparably, with neither a main effect of age (*F*_(1,20)_ = 0.53, *p *=* *0.48, η^2^ = 0.03) nor a significant interaction between age and trial type (*F*_(1,20)_ = 0.27, *p *=* *0.61, η^2^ = 0.01). There was, however, a main effect of trial type such that rats displayed significantly greater win–stay behavior than lose–shift behavior (*F*_(1,20)_ = 14,613.85, *p *<* *0.01, η^2^ = 1.00).

Reinforcement learning analysis of data from the reversal learning task revealed an increase in the learning rate parameter (α) across the eight sessions (Fig. 4*B*; main effect of session: *F*_(7,147)_ = 3.13, *p *<* *0.01, η^2^ = 0.09), but no difference between age groups (*F*_(1,12)_ = 0.08, *p *=* *0.78, η^2^ < 0.01) or interaction between age and session (*F*_(7,147)_ = 0.40, *p *=* *0.90, η^2^ = 0.01). An increase in learning rate across sessions can be interpreted as an improvement in knowledge of the environment, which allowed the rats to more rapidly update the lever values based on the probabilistic outcomes as the sessions progressed (i.e., they learned to learn more rapidly). Consistent with this finding, there was a significant decrease in the number of trials required to complete each reversal across the eight sessions (Fig. 4*C*; main effect of session: *F*_(7,147)_ = 7.90, *p *<* *0.01, η^2^ = 0.15), although neither the main effect of age (*F*_(1,21)_ = 0.92, *p *=* *0.35, η^2^ = 0.02) nor the age × session interaction (*F*_(7,147)_ = 1.84, *p *=* *0.08, η^2^ = 0.04) reached statistical significance. There was a modest but significant decrease in parameter β (choice stochasticity) across sessions (Fig. 4*D*; *F*_(7,147)_ = 2.18, *p *=* *0.04, η^2^ = 0.07), suggesting that rats sampled the levers more frequently as sessions progressed, possibly indicating greater awareness of rule changes (reversals); however, there was neither a main effect of age (*F*_(1,21)_ = 0.51, *p *=* *0.48, η^2^ < 0.01) nor an age × session interaction (*F*_(7,147)_ = 0.60, *p *=* *0.75, η^2^ = 0.02) on this measure.

### Progressive ratio schedule of reinforcement

**Figure 5.**
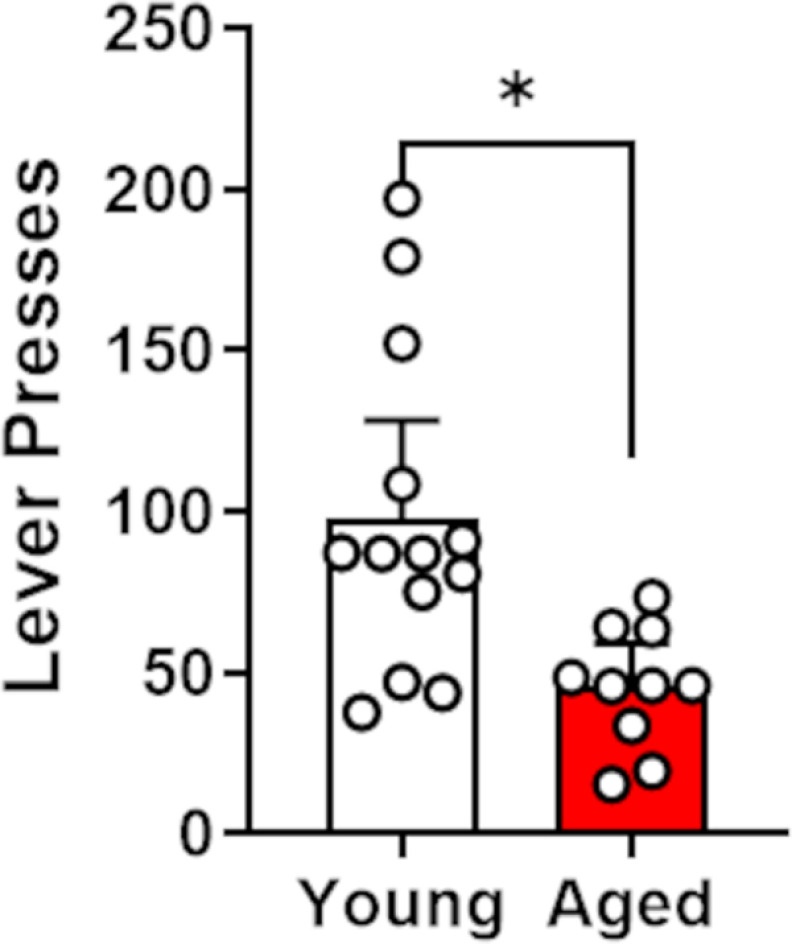
Performance on the progressive ratio schedule of reinforcement assay in young and aged rats. Young rats lever pressed significantly more than aged rats. Data are represented as the mean (±SEM) number of lever presses. *p ≤* *0.05.

Following the reversal learning task, rats (*n* = 13 young rats; *n* = 10 aged rats; from Cohorts 1 and 2 only) were tested on a progressive ratio schedule of reinforcement. Consistent with previous work in this rat strain (Hernandez et al., 2017), independent-samples *t*-tests revealed that aged rats made fewer lever presses (*t*_(21)_ = 3.48, *p *<* *0.01, *d* = 1.27), earned fewer food rewards (*t*_(21)_ = 3.77, *p *<* *0.01, *d* = 1.53), and had a lower breakpoint (*t*_(21)_ = 3.49, *p *<* *0.01, *d* = 1.42) than young rats (Fig. 5).

### Relationship between cognitive/behavioral measures

To evaluate relationships in performance among the behavioral tasks, correlations between behavior in the RDT (mean number of large, risky lever presses averaged across blocks 2–5 of stable performance), shock reactivity threshold assay, probabilistic reversal learning task (mean reversals completed across all eight sessions), and progressive ratio schedule of reinforcement (mean breakpoint ratio across all sessions) were examined. Spearman’s correlational analyses were conducted between variables separately for each age group. There was no relationship between performance in the RDT and any other behavioral measure in either age group (young rats, *p* values* *<* *0.20; aged, *p* values* *<* *0.23). In contrast, there were significant correlations between the breakpoint in the progressive ratio (PR) assay and the number of reversals in the probabilistic reversal learning task as well as the number of food pellets earned in the PR assay and the number of reversals in the probabilistic reversal learning task. These significant correlations, however, were limited to aged rats (aged rats: ρ = −0.64, *p *=* *0.05; young rats: ρ = −0.37, *p *=* *0.21) such that higher breakpoints and a larger number of earned food rewards (indicative of greater motivation to work for food) were associated with fewer reversals. This finding suggests that the moderate impairment in cognitive flexibility observed in the aged rats is unlikely to be related to a general reduction in motivation to work for food.

### Neuroimaging results

**Figure 6.**
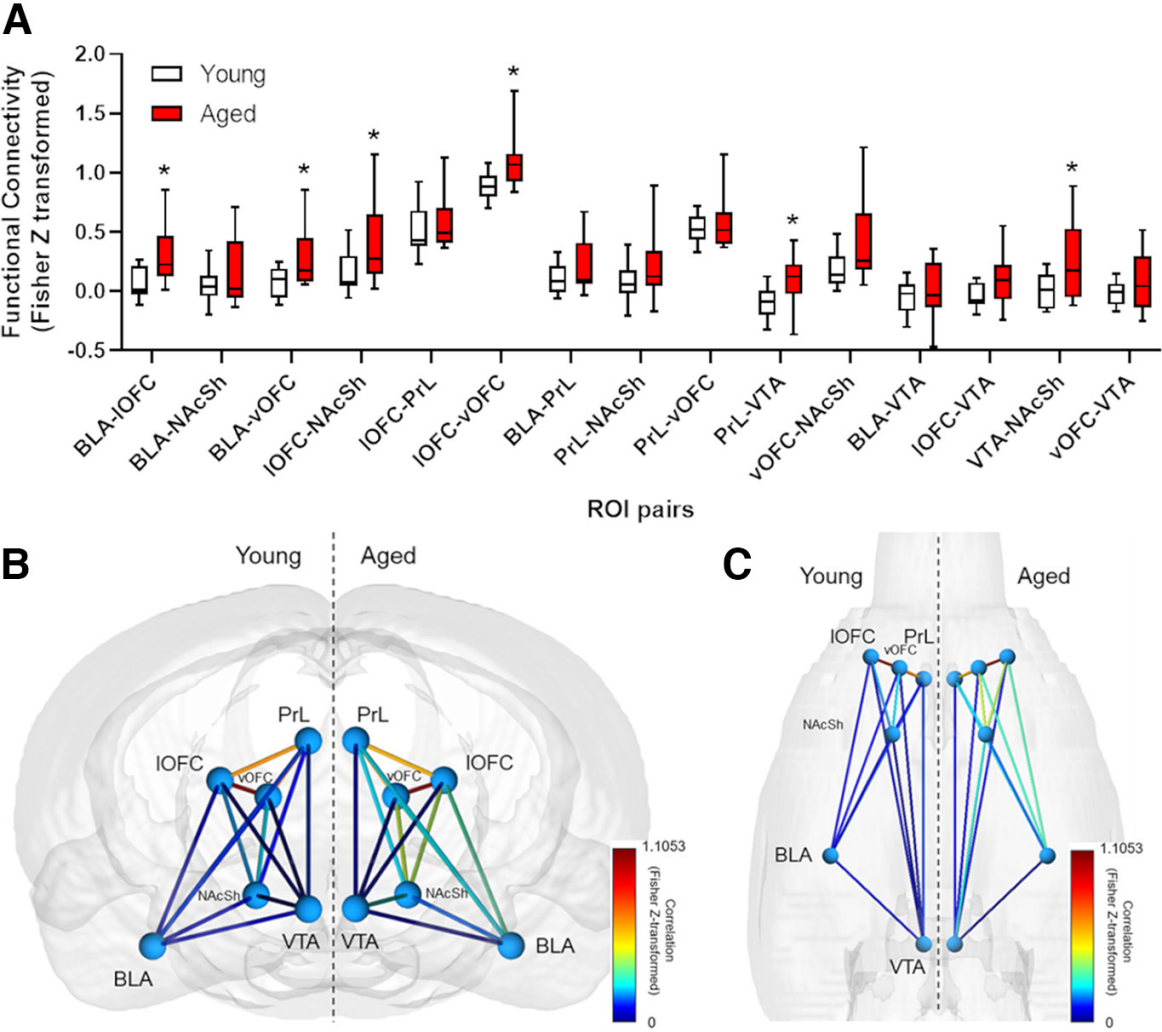
Functional connectivity between region-of-interest pairs in young and aged rats. Aged rats show greater resting-state functional connectivity than young rats among regions known to be critical to decision making under risk of punishment. ***A***, *Post hoc* analyses indicate significant differences between young and aged functional connectivity for specific region-of-interest pairs. Asterisks denote *p *≤* *0.05 after Bonferroni adjustment. ***B***, ***C***, Functional connectome maps of young (left) and aged (right) rats showing rostral-to-caudal (***B***) and ventral-to-dorsal (***C***) views of anatomically placed regions and their respective functional connections. Connectome maps were created with BrainNetViewer (Xia et al., 2013). Connecting “edges” are Fisher *z*-transformed Pearson’s *r* values.

A two-factor, repeated-measures ANOVA was used to evaluate age differences in functional connectivity in ROI pairs (age × ROI pair). This analysis revealed that the degree of connectivity varied between ROI pairs (main effect of ROI pair: *F*_(14,266)_ = 70.13, *p *<* *0.001, η^2^ = 0.57) and that across pairs, connectivity was greater in aged rats compared with young rats (main effect of age: *F*_(1,19)_ = 5.27, *p *=* *0.03, η^2^ = 0.05). To determine the source of this age difference, Bonferroni-adjusted *post hoc* analyses of functional connectivity were performed within each of the 15 ROI pairs. As depicted in Figure 6*A–C*, functional connectivity was greater in aged rats compared with young rats in the BLA–lOFC, BLA–vOFC, lOFC–NAcSh, lOFC–vOFC, PrL–VTA, and VTA–NAcSh pairs (*p* values* *<* *0.05). Additional analyses evaluated relationships between RDT performance and functional connectivity between each ROI pair in young adult and aged rats. As shown in Table 2, correlations between RDT performance and connectivity between the VTA and both OFC regions reached the *p* < 0.05 threshold in aged rats; however, these did not survive correction for multiple comparisons.

**Table 2:** Correlation coefficients comparing risky decision making performance and functional connectivity between region of interest pairs in young and aged rats

Region of interest pair	Group	Spearman’s ρ correlation coefficient	*p*-Value^†^
BLA.lOFC	Young	0.184	0.547
	Aged	0.1048	0.911
BLA.NAcSh	Young	0.173	0.571
	Aged	0.214	0.610
BLA.vOFC	Young	0.234	0.442
	Aged	0.024	0.955
lOFC.NAcSh	Young	0.234	0.442
	Aged	0.190	0.651
lOFC.PrL	Young	0.382	0.197
	Aged	0.095	0.823
lOFC.vOFC	Young	0.283	0.348
	Aged	−0.095	0.823
BLA.PrL	Young	0.492	0.087
	Aged	0.357	0.385
PrL.NAcSh	Young	0.377	0.204
	Aged	−0.095	0.823
PrL.vOFC	Young	0.228	0.453
	Aged	0.095	0.823
PrL.VTA	Young	−0.432	0.141
	Aged	−0.024	0.955
vOFC.NAcSh	Young	0.498	0.083
	Aged	0.381	0.352
BLA.VTA	Young	0.204	0.505
	Aged	0.476	0.233
lOFC.VTA	Young	0.470	0.105
	Aged	0.714	0.047*
VTA.NAcSh	Young	−0.030	0.922
	Aged	−0.429	0.289
vOFC.VTA	Young	0.083	0.789
	Aged	0.714	0.047*

*A significant correlation before corrections for multiple comparisons were made (*p *≤* *0.05).

† *p*-Values reported without multiple comparisons correction.

## Discussion

This study describes performance in aged and young adult rats in a decision making task involving the risk of explicit punishment, and how performance in this task is related to other cognitive/behavioral measures. The primary finding is that aged rats were more risk averse than young rats. In neither age group, however, was risk-taking behavior associated with shock sensitivity, motivation to work for food, or cognitive flexibility, nor did it correlate with functional connectivity between brain regions known to contribute to risk-based decision making. Despite a lack of a relationship with risk taking, functional connectivity among cortical and limbic structures was greater overall in aged rats relative to young rats. These findings largely corroborate those from studies in humans showing that risk taking decreases across the life span and additionally reveal that such reduced risk taking in aged rats is not attributable to age-related changes in sensitivity to footshock, motivation, or cognitive flexibility.

Reduced risk taking in aged rats in the RDT suggests that sensitivity to risk of punishment may increase with age. Contrary to the present findings, however, others have shown that older adults exhibit decreased sensitivity to punishment or loss. In a recent study, individuals across multiple age groups were tested in a probabilistic go/no-go monetary task in which they had to either respond or withhold a response to earn a monetary reward or to avoid punishment (loss of monetary reward). Older adults displayed poorer performance in this task, and reinforcement learning analyses revealed that this impairment was attributable to reduced sensitivity to both reward and punishment (Betts et al., 2020). These findings are consistent with those of Samanez-Larkin et al. (2007), who showed that older adults exhibit reduced negative arousal during anticipation of monetary losses. The “risk” in these studies, however, consisted of the potential loss of accrued monetary rewards in the task, rather than the risk of adverse and harmful consequences, such as loss of one’s own money or physical punishment (as in the RDT). Notably, in the present study there were no differences in shock reactivity thresholds between young and aged rats, indicating that increased sensitivity to the risk of punishment is not because of age-related changes in the perception of the footshock.

Differences in decision making between young and aged rats may arise as a result of age-related impairments in other cognitive or motivational domains. For example, previous work showed that among aged rats, less impulsive choice (greater preference for large, delayed over small, immediate rewards) is associated with reduced motivation to work for food, suggesting that an age-related reduction in incentive motivation could reduce the attraction of immediate rewards, rendering rats better able to delay gratification (Hernandez et al., 2017). Decreased preference for the large, risky reward in aged rats in the present study could thus be driven by similar impairments. Importantly, the age difference in progressive ratio performance is unlikely because of differential satiation between groups as only a few food pellets are earned in this task. In addition, it is unlikely that aged rats differed from young rats in reward perception, as both age groups preferred the large over the small reward to the same extent in the first block of trials in the RDT (when there was no risk of punishment). Moreover, the lower breakpoint of aged rats on the progressive ratio task is unlikely to reflect simply a reduction in willingness to pursue large, costly rewards in general, as aged rats, and particularly aged rats with low breakpoints on a progressive ratio task, are more likely than young rats to choose large rewards when they are accompanied by a delay to their delivery (Hernandez et al., 2017). Finally, there were no correlations between performance in the RDT and progressive ratio tasks in the present study. Considered together, these data suggest that although a reduction in food motivation in aged rats could render the large, risky reward less attractive, age differences in RDT performance are not likely attributable to differential satiation or reward perception/discrimination.

In addition to reduced motivation for food, aged rats displayed worse cognitive flexibility, or the ability to adapt to changes in task contingencies, in a probabilistic reversal learning task. It is conceivable that reduced preference for the large, risky reward in aged rats could be because of age-related cognitive inflexibility; however, the present data are incongruent with this interpretation. First, despite less flexible performance in the probabilistic reversal learning task, aged rats show a greater shift in their choice behavior across trial blocks in the RDT compared with young rats (Fig. 2*A*). Second, there were no correlations between performance in the RDT and the probabilistic reversal learning task in either age group. Hence, it is unlikely that reduced preference for the risky reward is due to impairments in cognitive flexibility. Although aged rats made fewer reversals than young rats in the probabilistic reversal learning task, the reinforcement learning analyses revealed no group differences in either learning rate (parameter α) or choice stochasticity (parameter β), indicating that aged and young rats learned and sampled the levers comparably when reversals occurred. Considered together, these findings suggest that there are only modest deficits in cognitive flexibility in aged rats relative to their younger counterparts. Age impairments in cognitive flexibility have been reported previously in deterministic reversal tasks in which a correct response is always rewarded (as opposed to probabilistic tasks, in which the correct response is more likely to be rewarded than the incorrect response). Similar to the data presented here, however, deficits were moderate and appeared in only a subset of aged rats (Barense et al., 2002; Schoenbaum et al., 2006; but see Beas et al., 2017); notably, such moderate deficits in cognitive flexibility contrast with findings in set-shifting tasks, in which age deficits are robust (Barense et al., 2002; Beas et al., 2013; Hernandez et al., 2017). In contrast to the current findings, however, a recent study reported that aged rats made more reversals than young rats in the same probabilistic reversal learning task used here (Tomm et al., 2018). It is unclear what factors might contribute to these discrepant findings, but one possibility is that rats in the current study underwent previous behavioral testing (i.e., the RDT) before testing in the probabilistic reversal learning task, whereas those in the study by Tomm et al. (2018) were behaviorally naive before reversal learning. Although all rats in the current study were retrained to press both levers comparably before progressing to the probabilistic reversal learning task, the previous lever associations in the RDT might have moderately interfered with learning new lever associations. Nevertheless, it appears that cognitive flexibility is not severely impacted and, under the appropriate conditions (i.e., Tomm et al., 2018), may even be enhanced in aging.

At the time of data collection for this study, only aged male rats of the Fischer 344 × Brown Norway F1 hybrid strain were available. Hence, the absence of female subjects is a limitation of this study, particularly in light of robust sex differences in the RDT as well as other decision making tasks involving punishment (Orsini et al., 2016, 2021; Orsini and Setlow, 2017; Chowdhury et al., 2019; Liley et al., 2019). In contrast to preclinical studies, there has been at least one study in humans that has examined sex differences in risk taking in aging. Using the Balloon Analogue Risk Task, Li et al. (2017) found that, consistent with the findings from the current study, older adults were more risk averse than young adults. Although there were gender differences in risk taking in young adults, these differences were absent in older adults (Li et al., 2017). It is therefore possible that sex differences in risk taking may dissipate with time, coinciding with age-related changes in neural activity that mediate risk taking and/or changes in gonadal hormones that mediate sex differences in risk taking in young adult rats (Orsini et al., 2021). Future studies will be needed to fully understand how risk-taking behavior changes with age across both sexes.

As mentioned previously, the type of risk that accompanies choices may determine how aging affects performance on decision making tasks. These different risks may account for at least some of the differences in the results of the present study compared with those from several previous studies of the effects of age on risky decision making. Gilbert et al. (2011) and others (Samson et al., 2015) found that aged rats performed no differently than young rats on a decision making task identical in design to the RDT but in which the risk was reward omission (i.e., a “probability discounting task”) rather than explicit punishment as in the RDT. In contrast, Tryon et al. (2020) found that aged rats were more inclined than young rats to choose small, guaranteed rewards over large, probabilistic rewards (i.e., aged rats were more risk averse than young rats), and that putative VTA dopamine neurons in aged rats were less responsive to rewards than those in young rats, implying a link between the activity of these neurons and risk behavior. Also relevant are separate findings that reductions in dopaminergic activity in the NAc (the target of many VTA neurons) appear to be predictive of less risk taking in young adult rats performing the RDT (Freels et al., 2020). Together, it is plausible that age-related reductions in VTA dopamine neuron activity (Branch et al., 2014; Tryon et al., 2020) play a causal role in driving more risk-averse behavior in aged rats. Notably, the idea that age-related reductions in dopamine neuron activity might lead to more risk-averse choices in the RDT appears to run counter to previous work showing that blockade of either D_1_ or D_2_ receptors has no effect on choice behavior, whereas activation of D_2_ (but not D_1_) receptors, both systemically and directly within NAc, induces risk aversion (Simon et al., 2011; Mitchell et al., 2014; Blaes et al., 2018). It is important to note, however, that aging affects multiple aspects of dopaminergic transmission. For example, the relatively larger decline in striatal D_1_ versus D_2_ receptor availability with age (Karrer et al., 2017) could shift the balance of activity toward D_2_ receptor signaling and greater risk aversion. Moreover, age-related reductions in VTA activity (and corresponding reductions in dopamine release) might favor signaling through higher-affinity D_2_ receptors over lower-affinity D_1_ receptors (Martel and Gatti McArthur, 2020).

A growing body of literature implicates dopamine neurons of the VTA as being critical for integrating aversive stimuli associated with decision making. These neurons are well known for their role in reward prediction error signaling (Schultz et al., 1997), wherein phasic increases in firing rate are linked to reward outcomes that are greater than expected. More recent studies show, however, that omission of an aversive stimulus also results in phasic increases in VTA dopamine neuron firing and have tied these increases to learning related to fear extinction and safety (Luo et al., 2018; Cai et al., 2020; Yau and McNally, 2022). Salinas-Hernández et al. (2018) further observed that optogenetic inhibition of VTA dopamine neurons concomitant with an omitted aversive stimulus impairs fear extinction learning. This suggests that age-related reductions in VTA dopamine neuron activity could underlie impaired fear extinction learning with age (Kaczorowski et al., 2012; Hernandez et al., 2022). In the case of probabilistic punishment as in the RDT, it is plausible that principles similar to fear extinction learning also apply to shock omission experienced in the RDT. If so, then the age-related risk aversion observed in this study may be explained by an inflation in the associative strength between the large lever press and the risk of punishment because of an impairment in learning during trials in which punishment is omitted. This theory could further explain the discrepancy between the effects of age on decision making involving the risk of reward omission (Gilbert et al., 2011; Samson et al., 2015) versus the risk of explicit punishment (present study), and suggests that aging more strongly affects VTA dopamine neuron activity tuned to punishment and its omission. Future studies in which dopaminergic neuron activity is assessed in aged rats during RDT performance are necessary to more directly address these issues.

Seed-based, resting-state functional connectivity (rsFC) analyses revealed increased functional connectivity in aged rats among select brain regions known to be involved in risky decision making. These findings are consistent with previous observations in rats of region-specific increases in rsFC with age after performance of a working memory task (Colon-Perez et al., 2019), and are further corroborated by studies in human subjects that observed increases in rsFC with age in long-range networks involving, but not limited to, the brainstem and amygdala (Biswal et al., 2010; Tomasi and Volkow, 2012). It has been suggested that greater functional connectivity implies some level of greater information transfer between regions (Sala-Llonch et al., 2015). A significant age-related increase in rsFC among brain regions belonging to an overall larger risky decision making network might therefore imply an association between age-related changes in rsFC and risky behavior. Indeed, the present study observed a number of correlations between rsFC in ROI pairs and RDT performance; however, these correlations did not survive correction for multiple comparisons (Table 2). Consequently, future studies should consider performing scans before and after behavioral characterization in the RDT to leverage the statistical power of within-subjects comparisons. By providing a baseline from which to compare rsFC of ROI pairs after behavioral training, such an experimental design would also address whether age differences in functional connectivity are because of overall age-related changes in neurobiology or are because of interactions between age and task experience.

Of the significant effects observed within the rsFC analyses, the BLA and OFC in particular were associated with several significant rsFC increases in the aged brain. In young adult rats, lesions of the OFC cause a reduction in risk taking in the RDT, whereas lesions of the BLA lead to an increase in risk-taking behavior (Orsini et al., 2015a). This work and others (Sias et al., 2021) suggest that these structures are likely critical to optimal decision making in the face of risk and punishment. Hence, increased functional connectivity with age between the BLA and OFC might indicate greater recruitment of and communication between these regions and their contributions to cognitive functions like decision making. As a result, the aged brain may overweight punishment and risks tied to rewards, promoting greater risk-averse behavior in aged rats compared with young. The fact that the BLA is critical for integrating emotional valence (Garavan et al., 2001) and biasing behavior away from risky choices (Orsini et al., 2015a) could imply that the aged BLA plays a greater role in risky decision making than in young rats. Indeed, in an intertemporal choice task designed similarly to the RDT, optogenetic inactivation of BLA during discrete timepoints within each decision trial revealed qualitative age differences in the degree to which BLA is engaged during different stages of the decision process (Hernandez et al., 2019), and similar temporally distinct roles for BLA are evident in the RDT (Orsini et al., 2017). On the other hand, there is also evidence for OFC dysfunction with age (Schoenbaum et al., 2006). Were the increased rsFC involving OFC indicative of dysfunction within this brain structure, it could contribute to the age-related reductions in risk taking observed here.

The current study also found a significant increase in rsFC between the VTA and PrL with age. Neurobiological changes in PrL and surrounding medial prefrontal cortex (mPFC) with age are associated with alterations in multiple executive functions, including impairments in set-shifting tasks assessing cognitive flexibility (Nicolle and Baxter, 2003; Beas et al., 2017; McQuail et al., 2021). In addition, age-related impairments in fear extinction learning (which could be viewed as a form of cognitive flexibility) are associated with *ex vivo* increases in PrL neuronal excitability (Vidal-Gonzalez et al., 2006; Kaczorowski et al., 2012). Increased rsFC between the PrL and VTA also corroborates work showing that the mPFC can regulate VTA activity (Carr and Sesack, 2000; Gao et al., 2007) and suggests that age-related changes in one region can cause dysregulation in both. Importantly, the PrL is directly involved in RDT performance through its role in adapting choice behavior in response to changing punishment probabilities across trials (Orsini et al., 2018)

In conclusion, the current study reveals age differences in decision making involving the risk of explicit punishment, with aged males exhibiting greater risk aversion than young males. Importantly, this age difference was not mediated by age-related differences in secondary factors, such as food motivation and shock sensitivity. Although decision making performance did not correlate with functional connectivity, functional connectivity was greater overall in aged rats relative to young rats among cortical and limbic brain regions. Coupled together, this information provides a foundation from which to explore the neural basis of age-related changes in decision making and identify strategies to preserve adaptive decision making abilities across the life span.

## Synthesis

Reviewing Editor: Christie Fowler, University of California, Irvine Decisions are customarily a result of the Reviewing Editor and the peer reviewers coming together and discussing their recommendations until a consensus is reached. When revisions are invited, a fact-based synthesis statement explaining their decision and outlining what is needed to prepare a revision will be listed below. The following reviewer(s) agreed to reveal their identity: Stan Floresco, Matthew Roesch.

Both reviewers felt that the manuscript and its main findings were of high interest with novel insights for the field. It was also noted that the use of additional tasks and functional scans provided more information, allowing for increased interpretation of the data.

Overall Assessment: This is an interesting study in which the authors investigated differences in risky decision making in aged male rats, using a now well-established discounting task involving choice between small reward and larger ones associated with probabilistic shock. Aged rats were more risk averse. The same animals were slightly impaired on a probabilistic reversal learning task and showed lower breakpoints when responding for food on a on a progressive ratio task, although these deficits could not explain the differences in risky decision making. Notably, aged rats showed no differences in shock sensitivity. The authors then used resting state fMRI to conduct functional connectivity analyses. Although there were no relationships between these measures and decision making, they did observe greater connectivity in certain systems in aged animals, including those with the VTA and forebrain and amygdala-OFC connectivity.

However both reviewers agreed that the manuscript could be strengthened as follows:

In general this is a fine study that adds to our understanding of how aging may alter decisions related to explicit punishments and the factors that may (or may not) contribute to these differences. A few, relatively minor comments/suggestions are intended to pull out more information from this data set and better integrate the findings with the literature.

1) I think the authors missed an opportunity to analyze and compare win-stay/lose-shift ratios on the RDT, and in particular compare these effects to the probabilistic reversal task. I understand that the authors conducted a more intricate reinforcement learning analysis on the latter task, but given that the authors ran animals on both tasks, it would be interesting to compare how these values relate across tasks and whether they differ between young and aged rats. Recent studies using the RDT have started to use these analyses, computing the probabilities of choosing the risky option after a non-punished risky choice or shifting to the safe option after a punished one. I would recommend the authors compute these values for the last few days of RDT training and probabilistic reversal training and compare them across groups and tasks. Indeed, a lack of effect on win-stay values on the RDT would further bolster their argument that the risk aversion observed is not due to motivational deficits.

2) The choice latency analysis is interesting, but why did the authors only compute this for forced choice trials. I think there would be value in looking at deliberation times during free choices and if there are any differences between groups there.

3) In comparing the behavioral results to the rsFC MRI analysis, the authors spend some time discussing the potential contribution of dopamine transmission, citing previous studies in aged rats. The impression left by the discussion is that age-related decreases in dopamine activity may contribute to these behavioral effects. However, I was surprised that the authors didn’t integrate information from previous studies showing that pharmacological reductions in dopamine do not alter choice on this task (whereas increased D2 receptor activity does lead to a risk averse choice profile). I think a few sentences in the discussion acknowledging these findings and integrating them with their thoughts on dopamine contribution would be useful.

## Author Response

Thank you very much for your email on October 21st regarding the reviews of our manuscript “Age-related changes in risky decision making and associated neural circuitry in a rat model” (eN-NWR-0385-22). Please find attached a revised version of this manuscript, in which we have incorporated the Reviewers’ comments and have noted modifications in red font. Below are responses that detail changes made to the manuscript that address these comments.

1) I think the authors missed an opportunity to analyze and compare win-stay/lose-shift ratios on the RDT, and in particular compare these effects to the probabilistic reversal task. I understand that the authors conducted a more intricate reinforcement learning analysis on the latter task, but given that the authors ran animals on both tasks, it would be interesting to compare how these values relate across tasks and whether they differ between young and aged rats. Recent studies using the RDT have started to use these analyses, computing the probabilities of choosing the risky option after a non-punished risky choice or shifting to the safe option after a punished one. I would recommend the authors compute these values for the last few days of RDT training and probabilistic reversal training and compare them across groups and tasks. Indeed, a lack of effect on win-stay values on the RDT would further bolster their argument that the risk aversion observed is not due to motivational deficits.

We agree with the Reviewers that win-stay/lose-shift ratios on the RDT are important behavioral outcomes to analyze. Although we included these in the original manuscript, we have now re-analyzed the data with a two-factor repeated measures ANOVA (rather than two separate t-tests as in the original manuscript), with trial type (win-stay vs. lose-shift) as the within-subjects factor and age as the between-subjects factor. Consistent with our original results, win-stay and lose-shift did not differ between aged and young rats. We have revised text in the Data Analysis section (pp. 9-10) of the manuscript to reflect the change in analysis and amended the text in the Results section to now include the results of this analysis (p. 14). Finally, we have included a figure depicting win-stay and lose-shift behavior in young and aged rats (Figure 2B).

The Reviewers’ comments also inspired additional analysis of the probabilistic reversal learning task. Specifically, we analyzed win-stay and lose-shift ratios, similar to those conducted by Dalton et al. (2014). In this task, these analyses provide information about how rats use information about a correctly rewarded choice (win-stay) vs. an incorrectly rewarded choice (lose-shift) to guide subsequent choices. The details of the analysis are now included in the Data Analysis section of the Methods (pp. 10-11) and the results are presented in the Results section (pp. 16-17).

2) The choice latency analysis is interesting, but why did the authors only compute this for forced choice trials. I think there would be value in looking at deliberation times during free choices and if there are any differences between groups there.

We appreciate this suggestion from the Reviewers and have now included the results from analysis of latencies to press levers during free choice trials. In contrast to latencies to press levers during forced-choice trials, there was no main effect of age on latencies to press levers during free choice trials [F (1, 25) = 1.38, p = 0.25, η2 = 0.05]. Additionally, there was no main effect of lever identity [F (1, 25) = 0.06, p = 0.80, η2 < 0.01] nor was there an interaction between age and lever identity [F (1, 25) = 1.46, p = 0.24, η2 = 0.06]. We have now included details about this analysis in the Materials and Methods section (p. 10) and the results of this analysis in the Results section (p. 15).

3) In comparing the behavioral results to the rsFC MRI analysis, the authors spend some time discussing the potential contribution of dopamine transmission, citing previous studies in aged rats. The impression left by the discussion is that age-related decreases in dopamine activity may contribute to these behavioral effects. However, I was surprised that the authors didn’t integrate information from previous studies showing that pharmacological reductions in dopamine do not alter choice on this task (whereas increased D2 receptor activity does lead to a risk averse choice profile). I think a few sentences in the discussion acknowledging these findings and integrating them with their thoughts on dopamine contribution would be useful.

The Reviewers make an excellent point, and we now provide discussion of this apparent discrepancy as well as a possible resolution on p. 22.

We thank the reviewers for their comments, which we believe have significantly improved the manuscript. Please let us know if we can provide any additional information.

Sincerely,

The Authors
